# Comparison of Clinical Efficiency and Safety Between Intra‐Articular Injection of Platelet‐Rich Plasma and Hyaluronic Acid for Hip Osteoarthritis: A Systematic Review and Meta‐Analysis

**DOI:** 10.1155/prm/6521682

**Published:** 2026-06-13

**Authors:** Di Zhang, Shen Hong Ma, He Ling Wang, Qiao Hua Han, Kai Shen, Weisheng Zhuang

**Affiliations:** ^1^ School of Rehabilitation Medicine, Henan University of Chinese Medicine, Zhengzhou, China, hactcm.edu.cn; ^2^ Department of Rehabilitation, Henan Provincial People’s Hospital, Zhengzhou, China, hnsrmyy.net; ^3^ School of Clinical Medicine, Henan University, Zhengzhou, China, henu.edu.cn

**Keywords:** hip osteoarthritis, meta-analysis, platelet-rich plasma

## Abstract

**Purpose:**

The aim of this study is to compare the clinical efficacy and safety of platelet‐rich plasma (PRP) and hyaluronic acid in the treatment of hip osteoarthritis.

**Methods:**

Data were collected from the PubMed, Embase, Cochrane Library, and Web of Science databases. The outcomes selected included the Visual Analog Scale (VAS) score, the Harris Hip Score (HHS), the Western Ontario, and McMaster Universities Osteoarthritis Index (WOMAC) and its relevant subscores. A standardized mean difference (SMD) was used as the combined effect size, and a random‐effects model was applied for analysis. The magnitude of the effect size was interpreted according to Jacob Cohen’s criteria.

**Results:**

A total of seven studies involving 465 patients were included. The meta‐analysis results indicated that in terms of pain relief, the PRP group showed a statistically significant advantage in VAS scores at 6 months (SMD = −0.38 and *p* = 0.01). Subgroup analysis revealed that LP‐PRP yielded significant improvement (SMD = −0.32 and *p* = 0.02), whereas LR‐PRP did not (SMD = −0.46 and *p* = 0.18). Similarly, multiple injections (SMD = −0.43 and *p* = 0.02) demonstrated superiority compared to a single injection (SMD = −0.12 and *p* = 0.63).Regarding WOMAC‐pain scores, the PRP group exhibited statistical differences at 6 months (SMD = −0.45 and *p* = 0.0006) and 12 months (SMD = −0.36 and *p* = 0.03). In terms of functional impairment, no significant differences were found in the HHS (SMD = 0.13 and *p* = 0.64) or WOMAC subscores (WOMAC‐stiffness: SMD = 0.01 and *p* = 0.97 and WOMAC‐function: SMD = −0.24 and *p* = 0.06) at any follow‐up time point. However, the WOMAC total score indicated that the overall health status of patients in the PRP group was superior to that in the HA group after treatment (SMD = −0.42 and *p* = 0.009).

**Discussion:**

The findings of this study indicate that compared with HA, PRP demonstrates a statistically significant advantage in alleviating pain (VAS and WOMAC‐pain) and improving overall symptoms (WOMAC total score) in patients with hip osteoarthritis (HOA) although the effect size was found to be moderate. However, regarding the improvement of joint function (HHS and WOMAC‐function), no significant differences were observed between the two groups. In summary, while PRP presents a statistical benefit over HA in the treatment of HOA, the effect size is limited; therefore, further high‐quality studies are warranted to definitively confirm its clinical superiority.

## 1. Background

Osteoarthritis (OA) is a common joint disorder, also referred to as a degenerative joint disease. It typically affects middle‐aged and older adults although younger individuals may also be affected, especially those with specific risk factors. OA primarily results from the degeneration and breakdown of articular cartilage, which increases friction between joint surfaces and ultimately leads to pain, swelling, stiffness, and functional impairment [1]. It can occur in various joints, including the hands, knees, hips, and spine. The etiology of OA is multifactorial, involving age, genetics, joint injuries, obesity, and lifestyle factors. With an aging global population and rising obesity rates, the prevalence of OA continues to increase.

Hip osteoarthritis (HOA) is one of the most common forms of OA, second only to knee OA. According to the World Health Organization, approximately 3.6% of the global population is affected by HOA, with the prevalence being even higher among individuals over the age of 65; moreover, men are affected more frequently than women [2]. The hip is one of the largest load‐bearing joints in the body, supporting body weight and enabling a wide range of movements. Because of the substantial mechanical stress placed on it, the hip joint is particularly prone to wear and degeneration. Symptoms of HOA include hip pain, stiffness, limited mobility, and impaired joint function. Patients often experience discomfort during daily activities such as walking, climbing stairs, and transitioning between sitting and standing. As the disease progresses, symptoms may worsen and significantly affect the quality of life [3].

Current treatment strategies for HOA primarily include pharmacological therapy, intra‐articular injections, physical therapy, and, in severe cases, surgical procedures such as total hip arthroplasty [4]. However, none of these approaches can fully cure HOA, and each carries potential side effects or risks. For example, the long‐term use of NSAIDs may cause gastrointestinal bleeding, corticosteroids (CSs) carry infection risk and may lead to osteonecrosis, and surgical procedures involve inherent perioperative risks. These limitations highlight the need for more effective and safer treatment options [5, 6].

Hyaluronic acid (HA), a major component of synovial fluid, is reduced in both concentration and molecular weight in OA. As a common intra‐articular therapy, HA enhances the viscoelasticity of synovial fluid, reduces friction between joint surfaces, alleviates inflammation, and promotes the synthesis of HA and proteoglycans by synovial cells [7]. A high‐quality systematic review reported that intra‐articular HA injections can effectively alleviate symptoms in HOA, particularly when high‐molecular‐weight hyaluronic acid (HMW‐HA) is used [8]. In 2000, the American College of Rheumatology (ACR) recommended HA injections as an analgesic option [9]. However, despite generally positive findings, research outcomes have been inconsistent and often fall short of expectations. Consequently, in 2013, the American Academy of Orthopaedic Surgeons (AAOS) withdrew its recommendation for HA use in OA management. Although HA remains more widely used than platelet‐rich plasma (PRP) or CSs, it does not repair damaged joint tissues and is limited in its capacity to support structural regeneration [10]. Therefore, identifying novel therapies capable of promoting tissue repair is of high clinical importance.

PRP injection is an emerging therapy classified under bioactive regenerative medicine. Its mechanism is rooted in the biological properties of platelets and the regenerative activity of the growth factors they release, which activate the body’s natural healing cascade to promote tissue repair and regeneration [11, 12]. Growth factors in PRP stimulate gene expression, induce mRNA transcription, and support the synthesis of proteins required for tissue repair, thus promoting cell proliferation, differentiation, angiogenesis, and extracellular matrix production. These growth factors interact within a complex regulatory network, working synergistically to enhance tissue regeneration and ultimately facilitate healing of damaged structures [13, 14]. In addition, PRP contains abundant leukocytes that help remove pathogens and necrotic tissues. Upon activation (e.g., with thrombin), high levels of antimicrobial peptides are released, which not only possess endogenous antibiotic properties but also exert immunomodulatory effects, substantially enhancing local antimicrobial capacity [14].

PRP therapy offers several advantages, including high safety and minimal invasiveness. Because it is derived from a patient’s own blood, the risks of disease transmission and immunological rejection are avoided [15]. As a nonoperative approach, PRP does not require immobilization after injection; patients may resume normal activities after a brief rest, thereby avoiding surgical risks and long recovery periods. In recent years, PRP has been widely applied across multiple medical fields, including wound repair, esthetic medicine, dentistry, and diabetic foot care [16]. It is also used as an adjunct to surgery, promoting incision healing and reducing postoperative complications.

Despite increasing interest in PRP as a potential treatment for HOA, a clear consensus regarding its comparative effectiveness relative to HA is still lacking. Moreover, the relative efficacy rankings of PRP, HA, and other injectables may vary depending on evaluation time points and outcome measures, indicating the absence of a single optimal injection therapy for HOA. Therefore, in this study, we systematically searched multiple databases for recent research directly comparing PRP and HA in the treatment of HOA, with the aim of evaluating the therapeutic value of PRP and providing more reliable and clinically relevant guidance for injection‐based management of HOA.

## 2. Materials and Methods

### 2.1. Register

This review was conducted following the guidelines outlined in the Cochrane Handbook and reported according to the PRISMA statement. It was also preregistered in PROSPERO (reference number: CRD42024561683).

### 2.2. Literature Search

The literature search was conducted across several databases, including PubMed, Embase, Cochrane Library, and Web of Science. The search covered studies from the inception of each database up to July 11, 2025. The search strategy used was (((“Platelet‐Rich Plasma”[Mesh]) OR (Plasma, Platelet‐Rich[Title/Abstract])) OR (Platelet Rich Plasma[Title/Abstract])) AND (((((((“Osteoarthritis, Hip”[Mesh]) OR (Hip Osteoarthritis[Title/Abstract])) OR (Osteoarthritis Of Hip[Title/Abstract])) OR (Osteoarthritis Of Hips[Title/Abstract])) OR (Coxarthrosis[Title/Abstract])) OR (Coxarthroses[Title/Abstract])) OR (Osteoarthritis of the Hip[Title/Abstract])).

### 2.3. Inclusion and Exclusion Criteria

Inclusion criteria are as follows: (1) randomized controlled trials (RCTs); (2) study population consisting of patients with HOA; (3) intervention involving intra‐articular injection of PRP or HA therapy; and (4) outcome measures that include at least Visual Analog Scale (VAS) or McMaster Universities Osteoarthritis Index (WOMAC)‐pain.

Exclusion criteria: (1) studies with incomplete data that cannot be analyzed; (2) studies for which the full text is not available; (3) duplicated studies; and (4) nonrandomized controlled trials.

### 2.4. Literature Screening and Data Extraction

Two researchers independently screened the literature and extracted the following data from the included studies: author, publication year, sample size, mean age, intervention methods, outcome parameters, and follow‐up duration. The efficacy indicators primarily focused on outcome measures at follow‐up times ranging from one month to twelve months, including VAS, Harris Hip Score (HHS), WOMAC, WOMAC pain, WOMAC stiffness, and WOMAC functional limitation. Data collection or subgroup analysis should be conducted based on the follow‐up time points of the original studies. In case of any disagreements, a third researcher was consulted for arbitration. After reaching consensus, the data were entered into RevMan 5.4 for analysis.

### 2.5. Quality Assessment

The methodological quality of each included study was assessed using the Cochrane Collaboration tool. According to this tool, studies were classified as having “low,” “unclear,” or “high” risk for the following types of bias: selection bias, allocation bias, performance bias, reporting bias, attrition bias, and other bias.

### 2.6. Statistical Analysis

Meta‐analyses were performed using RevMan5.4. Given the variations in measurement scales across the included studies, the standardized mean difference (SMD) with 95% confidence intervals (95%CIs) was utilized as the pooled effect size for all continuous outcomes. The magnitude of the effect size was interpreted according to Cohen’s criteria [17], where an SMD of 0.2 represents a small effect, 0.5 a medium effect, and 0.8 a large effect. The DerSimonian–Laird (DL) method was employed to estimate the between‐study variance (*τ* ^2^), and the Wald method was applied to calculate the 95% CIs for the pooled effect sizes. A *p* value of < 0.05 was considered statistically significant. Furthermore, given the limited number of included studies, a sensitivity analysis was conducted using the “leave‐one‐out” method to assess the robustness of the pooled results and to investigate potential sources of heterogeneity.

### 2.7. Clinical Significance Threshold Reference

For clinical understanding, the discussion section refers to the minimal clinically important difference (MCID) thresholds from previous studies for the original outcome measures, including VAS [18] (0.9 cm or 9 mm, based on a 0–10 cm scale), WOMAC total score [19] (6.6 points, based on a 0–100 scale), and WOMAC‐pain [20] (0.75 points, based on a 0–10 scale).

## 3. Outcome

### 3.1. Literature Results

**FIGURE 1:**
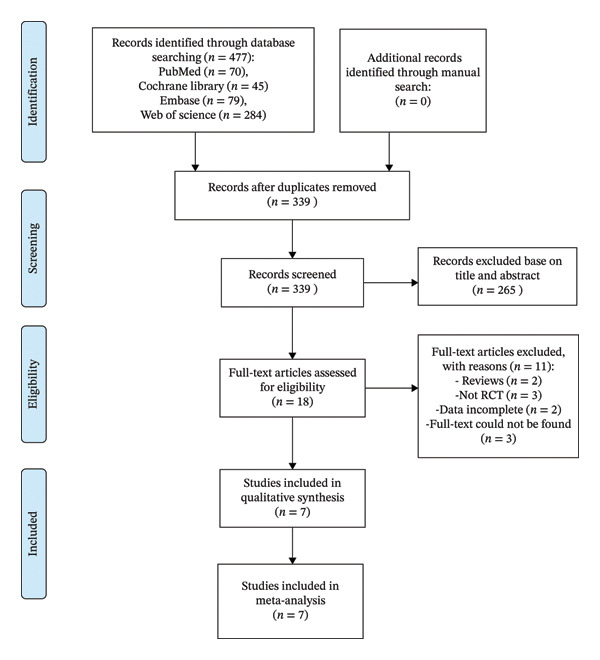
The flowchart of the literature search and screening process.

A total of 477 articles were retrieved, and after removing duplicates, 339 articles remained. After reading and screening the titles and abstracts, 18 studies were initially identified. Following a detailed full‐text review, 7 studies were ultimately included (Figure 1). All 7 studies were randomized controlled trials, with participants divided into an experimental group and a control group. The experimental group received autologous PRP injections, while the control group was treated with HA. The severity of arthritis in the included patients ranged from Kellgren–Lawrence (K–L) Grade I to Grade IV (Table 1).

**TABLE 1:** Basic information of the included studies.

Author	Country	Year	Group	Sample size	K–L scale	Intervention (mL)	Frequency	PLT Conc.	Leukocyte	Activator	Age	Male [n (%)]	Follow‐up (m)	Outcomes
Villanova‐López	Spain	2019	PRP	34	I–IV	6	1/wk × 1	390%	LP‐PRP	CaCl2	61.2 ± 9.72	14 (41.2)	12	VAS, HHS, WOMAC
Battaglia	Italy	2013	PRP	50	II–IV	5	1/2 wk × 3	600%	LR‐PRP	CaCl2	51 ± 12	30 (60)	12	VAS, HHS
Dallari	Italy	2016	PRP	44	I–IV	5	1/wk × 3	NS	NS	CaCl2	NS	20	12	VAS, WOMAC
Di Sante	Italy	2016	PRP	21	II–III	4	1/wk × 3	100%–150%	LP‐PRP	NS	71.37 ± 6.03	11	4	VAS, WOMAC
Doria	Italy	2017	PRP	40	0–II	5	1/wk × 3	NS	LR‐PRP	NS	67.3 ± 5.8	NS	12	VAS, HHS, WOMAC
J. Kraeutler	America	2021	PRP	19	II‐III	1	1/wk × 3	200%–300%	LP‐PRP	CaCl2	53.3 ± 8.4	8 (42.1)	24	WOMAC
Nouri	Iran	2022	PRP	32	II‐III	5–6	1/2 wk × 2	550%	LP‐PRP	None	58.22 ± 5.10	10 (31.3)	6	VAS, WOMAC

### 3.2. Literature Quality Evaluation Results

**FIGURE 2:**
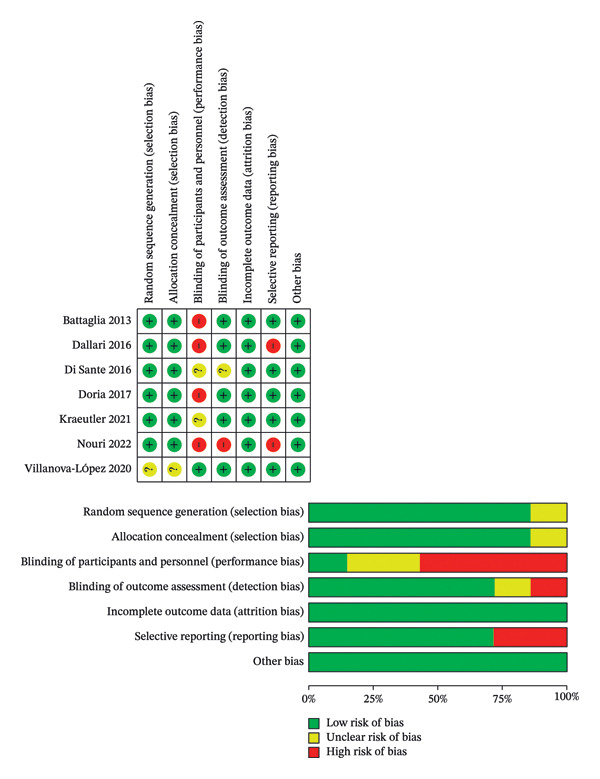
Risk of bias summary of RCTs.

The Cochrane Risk of Bias tool was used for an independent assessment of the included studies. Among these, one study described randomization but did not specify the randomization method or allocation process, which was categorized as “uncertain risk.” Regarding blinding, four studies did not blind the participants or primary researchers, which was categorized as “high risk,” and two studies did not provide details, categorized as “uncertain risk.” One study’s outcomes may have been influenced by the lack of blinding, which was categorized as “high risk.” Another study did not specify whether the outcomes were evaluated using blinding, categorized as “uncertain risk.” Two studies reported outcome indicators that were inconsistent with the preset criteria, categorized as “high risk.” No other risks were identified (Figure 2).

**FIGURE 3:**
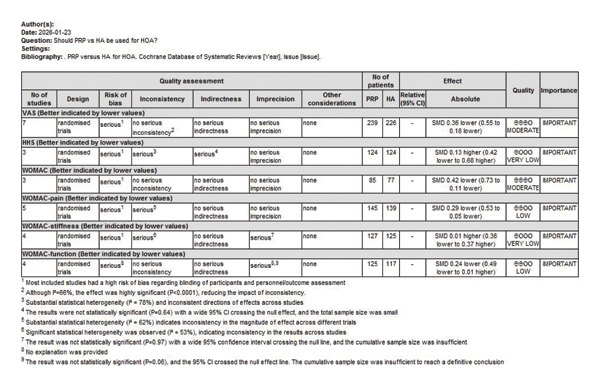
GRADE assessment of the certainty of evidence for each outcome measure.

The certainty of evidence for the outcome measures across the seven included studies was evaluated using the GRADE approach. Due to limitations in the implementation of blinding (risk of bias), the overall certainty of the evidence ranged from “moderate” to “very low.” Specifically, the evidence for VAS scores and WOMAC total scores was graded as “moderate,” while WOMAC‐pain and WOMAC‐function were rated as “low.” The evidence for HHS and WOMAC‐stiffness was classified as “very low.” These assessments are summarized in Figure 3.

### 3.3. Study Results

VAS and WOMAC scores were selected as the primary outcomes, while other measures were considered secondary outcomes.

### 3.4. VAS

**FIGURE 4:**
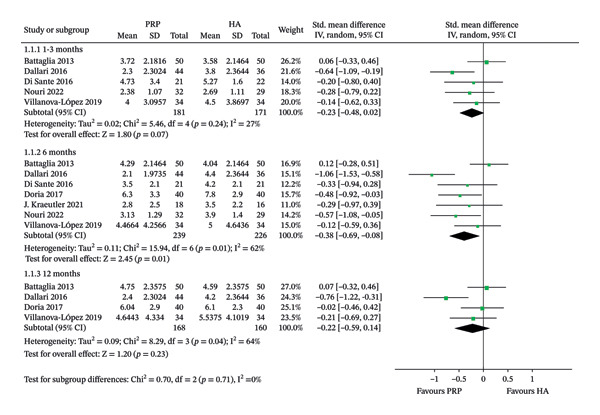
The forest plot of VAS scores between the PRP group and HA group.

Seven studies [21–26] utilized the VAS score as an outcome measure to compare PRP with HA. As illustrated in Figure 4, the PRP group exhibited significantly lower VAS scores compared with the HA group at 6 months posttreatment (SMD = −0.38, 95% CI [−0.69, −0.08], *p* = 0.01; *τ* ^2^ = 0.11, *I* ^2^ = 62%, and *p* = 0.01), representing a moderate effect size according to Cohen’s criteria. However, no significant differences were observed between the two groups at 3 months (SMD = −0.23, 95% CI [−0.48, 0.02], *p* = 0.07; *τ* ^2^ = 0.02, *I* ^2^ = 27%, and *p* = 0.24) or 12 months (SMD = −0.22, 95% CI [−0.59, 0.14], *p* = 0.23; *τ* ^2^ = 0.09, *I* ^2^ = 64%, *p* = 0.04).

**FIGURE 5:**
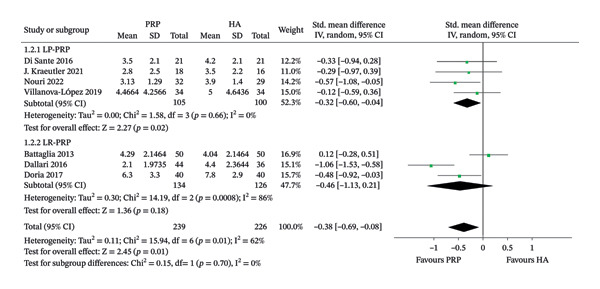
Subgroup analysis of VAS scores stratified by the PRP leukocyte content.

**FIGURE 6:**
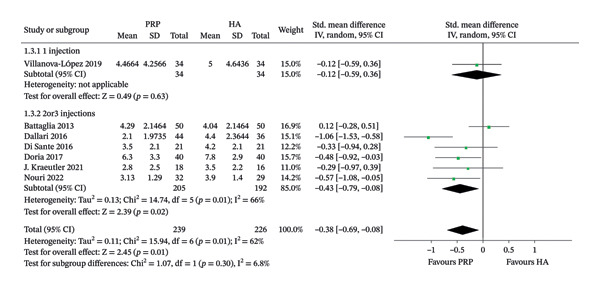
Subgroup analysis of VAS scores stratified by injection frequency.

Subgroup analyses revealed that leukocyte‐poor PRP (LP‐PRP) yielded a statistically significant improvement (SMD = −0.32, 95% CI [−0.60, −0.04], *p* = 0.02; *τ* ^2^ = 0, *I* ^2^ = 0%, and *p* = 0.66), whereas leukocyte‐rich PRP (LR‐PRP) did not (SMD = −0.46, 95% CI [−1.13, 0.21], *p* = 0.18; *τ* ^2^ = 0.3, *I* ^2^ = 86%, and *p* = 0.0008] (Figure 5). Similarly, multiple injections (SMD = −0.43, 95% CI [−0.79, −0.08], *p* = 0.02; *τ* ^2^ = 0.13, *I* ^2^ = 66%, and *p* = 0.01) resulted in significantly lower VAS scores, while single injections did not show a significant difference (SMD = −0.12, 95% CI [−0.59, 0.36], and *p* = 0.63) (Figure 6). Regarding the sensitivity analysis for the 1–3‐month subgroup, excluding the study by Battaglia et al. shifted the result from nonsignificant (*p* = 0.07) to statistically significant (*p* = 0.008), with heterogeneity decreasing from 27% to 0%. The results of the sensitivity analyses for the other two subgroups remained robust and unchanged.

### 3.5. WOMAC

**FIGURE 7:**
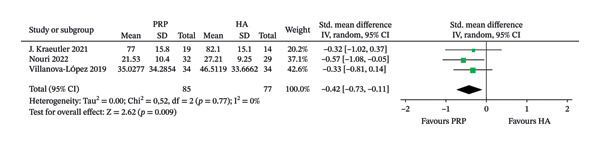
The forest plot of WOMAC scores between the PRP group and HA group.

Three studies [25–27] utilized the WOMAC total score as an outcome measure to evaluate posttreatment outcomes between the two groups. The meta‐analysis results demonstrated that the WOMAC scores in the PRP group were significantly lower than those in the HA group (SMD = −0.42, 95% CI [−0.73, −0.11], and *p* = 0.009), with no significant heterogeneity observed (*τ* ^2^ = 0.00, *I* ^2^ = 0%, and *p* = 0.77). The statistical effect size was considered moderate. Furthermore, sensitivity analysis did not alter the results, indicating that the conclusion remains robust (Figure 7).

### 3.6. WOMAC‐Pain

**FIGURE 8:**
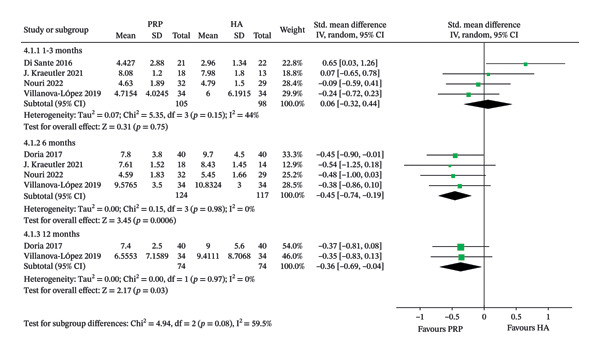
The forest plot of WOMAC‐pain scores between the PRP group and HA group.

Five studies [23–27] reported WOMAC pain scores following intervention in both the PRP and HA groups. The results indicated that the PRP group significantly outperformed the HA group at 6 months (SMD = −0.45, 95% CI [−0.71, −0.19], *p* = 0.0006; *τ* ^2^ = 0, *I* ^2^ = 0%, and *p* = 0.98) and 12 months (SMD = −0.36, 95% CI [−0.69, −0.04], *p* = 0.03; *τ* ^2^ = 0, *I* ^2^ = 0%, and *p* = 0.97), representing a moderate effect size. In contrast, no significant difference was observed at 1–3 months (SMD = 0.06, 95% CI [−0.32, 0.44], *p* = 0.75; *τ* ^2^ = 0.07, *I* ^2^ = 44%, and *p* = 0.15). Sensitivity analysis via the “leave‐one‐out” method confirmed that the conclusions remained consistent and robust, with no qualitative changes observed (Figure 8).

### 3.7. WOMAC‐Stiffness

**FIGURE 9:**
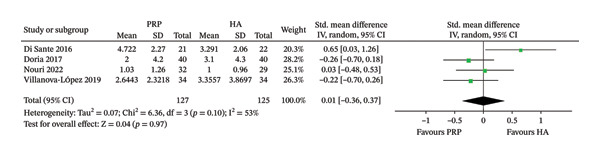
The forest plot of WOMAC‐stiffness scores between the PRP group and HA group.

Four studies [23–26] presented posttreatment WOMAC stiffness scores. The analysis revealed no statistically significant difference between the PRP and HA groups (SMD = 0.01, 95% CI [−0.36, 0.37], *p* = 0.97; *τ* ^2^ = 0.07, *I* ^2^ = 53%, and *p* = 0.1). Furthermore, sensitivity analysis yielded consistent results, confirming the stability of these findings (Figure 9).

### 3.8. WOMAC‐Function

**FIGURE 10:**
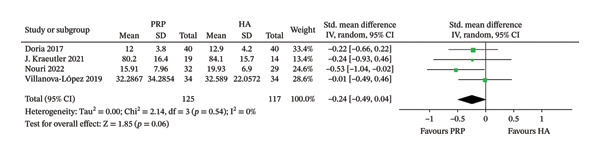
The forest plot of WOMAC‐function scores between the PRP group and HA group.

Four studies [24–27] utilized the WOMAC function subscale to compare therapeutic efficacy between the two groups. The analysis yielded negative results, indicating no statistically significant difference between the PRP and HA groups (SMD = −0.24, 95% CI [−0.49, 0.01], *p* = 0.06; *τ* ^2^ = 0, *I* ^2^ = 0%, and *p* = 0.54). Notably, however, when the study by Villanova‐López et al. was excluded during sensitivity analysis, the pooled result shifted from nonsignificant (*p* = 0.06) to statistically significant (*p* = 0.03) (Figure 10).

### 3.9. HHS

**FIGURE 11:**
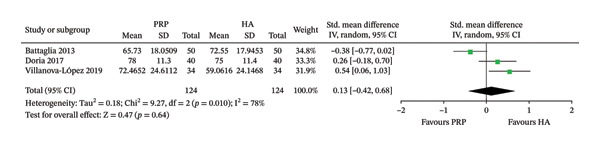
The forest plot of HHS scores between the PRP group and HA group.

Three studies [21, 24, 26] utilized the HHS as an outcome measure to evaluate treatment efficacy. The initial analysis showed no statistically significant difference between the PRP and HA groups (SMD = 0.13, 95% CI [−0.42, 0.68], and *p* = 0.64), with high heterogeneity observed (*τ* ^2^ = 0.18, *I* ^2^ = 78%, and *p* = 0.010). However, sensitivity analysis indicated that these findings were highly unstable. Specifically, after excluding the study by Battaglia et al., heterogeneity dropped drastically from 78% to 0%, and the pooled result shifted from nonsignificant to statistically significant (*p* = 0.02) (Figure 11).

### 3.10. Safety

Among the seven included studies, four [23, 24, 26, 27] did not report any adverse events or complications. Reported adverse events in both the PRP and HA groups were primarily characterized by self‐limiting post‐injection pain. Specifically, the PRP group reported 13 cases of aggravated local pain or joint stiffness, while the HA group reported 10 cases of similar symptoms and 2 cases of skin reactions. All symptoms resolved spontaneously within 48 h, and no serious adverse events (SAEs), such as severe infections or nerve injuries, occurred (Table 2).

**TABLE 2:** Adverse effects and complications that emerged in the included studies.

Adverse effects and complications	Studies
No adverse reactions and complications were present or documented	Dallari, Di Sante, Kraeutler, Villanova
Pain during or after injection	Battaglia, Doria, Nouri
Superficial hematoma	Battaglia
Symptoms such as fever, stiffness, heaviness, etc.	Nouri

## 4. Discussion

With the intensifying trend of global population aging, HOA has increasingly emerged as a prevalent condition, posing significant challenges to clinicians [28]. The findings of this meta‐analysis suggest that PRP may offer superior efficacy compared to HA in alleviating pain (VAS and WOMAC‐pain) and improving overall symptoms (WOMAC total score) although no significant differences were observed regarding functional improvement (HHS and WOMAC‐function). While a recent study by Migliorini et al. [8] reported results contrary to ours—finding high molecular weight HA superior to a mixed control group of PRP and CSs—this discrepancy likely stems from the confounding design of their control group. Since the therapeutic benefits of CSs typically persist for a maximum of 12 weeks [29], their short‐term efficacy complicates and potentially hinders a precise direct comparison between PRP and HA.

Consistent with our findings, Samble et al. [30] also observed that PRP might be superior to HA in pain relief at the 6‐month follow‐up, despite inconsistencies in functional outcomes. Similarly, Belk et al. [31] noted that while both therapies yielded significant symptomatic improvements, no significant statistical difference was observed in the short term. Although a separate network meta‐analysis [32] comparing multiple injectables found no intervention significantly superior to placebo, such indirect comparisons may dilute the direct therapeutic effects of specific interventions. Furthermore, treating PRP as a homogeneous intervention without accounting for variations in preparation lacks methodological rigor. Therefore, building upon previous research, the present study focuses exclusively on the direct comparison between PRP and HA. By performing detailed analyses stratified by follow‐up duration and PRP composition and by incorporating the most recent RCTs, this study aims to further refine the evidence regarding the clinical efficacy of PRP versus HA in the treatment of HOA.

### 4.1. Pain Relief

Hip pain constitutes the primary symptom of HOA and remains the predominant reason for patients seeking medical intervention. Multiple studies have demonstrated that while both PRP and HA are effective in alleviating HOA‐related pain, they differ significantly in their onset of action and duration of therapeutic effect [22, 25, 33]. Although PRP is generally considered safe, recommendations for its use in HOA remain controversial due to the lack of standardized preparation and injection protocols, as well as the paucity of robust evidence regarding its long‐term benefits [34].

In the present study, we observed that PRP demonstrated a modest superiority in overall pain relief, particularly at 6 months (VAS SMD = −0.38) and between 6 and 12 months posttreatment (WOMAC‐pain SMD = −0.45 and −0.36, respectively). This suggests that the analgesic advantage of PRP may require several months to manifest. Furthermore, subgroup analysis indicated that the leukocytes present in LR‐PRP might exacerbate inflammatory responses, rendering its pain‐relieving effect less significant than that of LP‐PRP; additionally, multiple injections appeared superior to single administration. Similarly, Bennell et al. [35] noted that while PRP provides short‐term symptomatic relief and functional improvement, the stability of its efficacy is likely closely correlated with patient age, the severity of degeneration, and the duration of the intervention. Crucially, despite the statistical superiority and moderate effect size of the PRP group, a distinction must be drawn between statistical significance and clinical relevance. Given the subjective nature of scoring systems like VAS, the concept of the MCID is central to assessing the patient’s perceived benefit. This implies that in clinical practice, a statistically significant improvement (*p* ≤ 0.05) does not necessarily translate into a tangible clinical benefit perceived by the patient; therefore, caution is warranted when advocating for PRP as the preferred first‐line treatment option.

### 4.2. Functional Improvement

In addition to pain control, the recovery of joint function is another key indicator of the effectiveness of HOA treatment. Functional impairment directly compromises patients’ quality of life and mobility, particularly as the disease progresses to moderate‐to‐advanced stages, where restrictions in range of motion and muscle weakness become increasingly pronounced [36]. Consequently, effective treatment strategies should extend beyond mere analgesia to actively promote the restoration of joint range of motion, gait stability, and activities of daily living.

In the present study, although PRP demonstrated a statistical advantage in pain alleviation, improvements in functional scores, such as the HHS, were not statistically significant. Functional impairment in HOA is frequently accompanied by irreversible structural alterations. This is particularly evident in patients with severe disease (K–L Grades III‐IV), characterized by joint space narrowing, osteophyte formation, subchondral bone damage, and capsular contracture, all of which mechanically restrict joint mobility. Even if PRP injection confers a biological analgesic effect, such mechanical and physical structural damage may be difficult to reverse solely through intra‐articular injections [37]. While the WOMAC total score was the only functional metric to exhibit a statistical difference, the effect size was merely moderate (SMD = −0.42), and its true clinical relevance remains to be validated.

Similarly, Santiago et al. [38] noted that the combination of PRP and HA failed to demonstrate the anticipated synergistic functional benefits. This observation aligns with the instability of the HHS outcomes observed in our study although whether combined therapy follows the same efficacy trajectory as monotherapy remains to be confirmed. This suggests that while the bioactivity of PRP may be sufficient to modulate pain signaling pathways or mitigate mild inflammatory responses, it is likely insufficient to reverse the severe anatomical structural damage responsible for functional impairment.

Although some studies have indicated that PRP can improve joint function to a certain extent—particularly in patients with early‐stage HOA (K–L Grades I‐II), where it positively impacts activities of daily living and weight‐bearing lower limb coordination [22, 27, 39]—the high heterogeneity and strict inclusion criteria of these studies (which predominantly enrolled younger patients with milder symptoms) do not definitively support the conclusion that PRP is significantly superior to HA or placebo. Therefore, we maintain that in the absence of high‐quality clinical studies with large sample sizes and long‐term follow‐up, there is insufficient evidence to conclude that PRP offers superior functional improvement compared to standard treatments.

### 4.3. Analysis of Clinical Heterogeneity and Assessment of Robustness

To deeply investigate the sources of heterogeneity, this study employed a combination of sensitivity and subgroup analyses. The sensitivity analysis revealed that for VAS (1–3 months) and HHS, excluding the study by Battaglia et al. shifted the pooled results from nonsignificant to statistically significant (*p* < 0.05), accompanied by a substantial reduction in heterogeneity (I^2^ = 0%). This discrepancy may be attributed to the use of HMW‐HA in the control group of that study; given that HMW‐HA possesses superior short‐term analgesic and lubricating properties [29], its use may have obscured the comparative differences between PRP and HA. Furthermore, the exclusion of the study by Villanova‐López—the sole trial utilizing a single‐injection protocol—resulted in a more pronounced difference between the two groups (*p* < 0.05), implying that injection frequency exerts a potential influence on therapeutic outcomes. Consequently, we conducted subgroup analyses of VAS scores stratified by different administration protocols to identify potential factors affecting clinical significance. The results confirmed that injection frequency and leukocyte concentration were the primary sources of the observed heterogeneity. Multiple‐injection protocols not only demonstrated significant efficacy (*p* = 0.02) but also yielded a robust effect size (SMD = −0.43), suggesting that for the hip joint—a large‐volume, metabolically active articulation—a single injection may fail to sustain the bioactive concentration required to trigger tissue repair mechanisms. Concurrently, LP‐PRP exhibited high consistency (*I* ^2^ = 0%) at 6 months and was significantly superior to HA (*p* = 0.02), whereas the high leukocyte content in LR‐PRP may induce short‐term local inflammatory reactions, leading to fluctuations in pain scores. Therefore, a regimen combining multiple injections with LP‐PRP appears to represent a more predictable and robust therapeutic option.

### 4.4. Safety Profile

As two common conservative therapeutic modalities for HOA, both PRP and HA are widely accepted due to their minimally invasive nature, localized application, and high repeatability. However, across the seven included studies, the reporting of adverse events exhibited severe inconsistency and incompleteness. Although some studies reported “no observed adverse events,” this likely reflects disparities in surveillance mechanisms or underreporting rather than a genuine absence of risk, thereby potentially confounding the conclusion regarding the favorable safety profile of PRP. Given the already insufficient sample size, the data from the mere three studies reporting adverse events are inadequate to discern any distinct safety differences between the two groups. Previous research suggests that adherence to standard aseptic protocols, appropriate needle selection, and precise injection site localization are technical factors that may contribute to mitigating the risk of adverse reactions [40]. Therefore, it is imperative that future studies adopt a more standardized consensus on safety reporting and utilize larger sample sizes to provide more reliable evidence for safety assessment.

### 4.5. Limitations of the Study

1. Heterogeneity of PRP protocols: As outlined in Table 1, the included studies exhibited substantial variability in PRP preparation and application, including differences in leukocyte content, platelet concentration (ranging from 1.5 to 6 times baseline), the use of activators, and injection protocols. Although subgroup analyses were conducted regarding leukocyte content and injection frequency, the high biological inconsistency of PRP products remains a primary source of heterogeneity. Consequently, disregarding this “class effect” during comparative analysis may be methodologically unsound.
2. Risk of bias and blinding issues: The majority of studies failed to implement complete blinding for subjects and outcome assessors. Given the highly subjective nature of scoring systems such as VAS and WOMAC, patients aware of their group allocation may experience a placebo effect, particularly regarding PRP, which is widely marketed as an “advanced therapy.” Similarly, assessors may subconsciously assign more positive evaluations to the PRP group. This creates a risk that the pooled effect sizes in this study are overestimated, thereby compromising the robustness of the overall evidence. Therefore, when interpreting clinical benefits, it must be acknowledged that these statistical differences may partially stem from data bias inherent in nonblinded designs.
3. K–L staging: The included studies encompassed a broad patient population ranging from K–L Grades 0 to IV (Table 1). Theoretically, early‐stage OA may respond more favorably to biological therapies; however, the primary studies did not provide detailed outcome data (mean/SD) stratified by the K–L grade. Consequently, we were unable to conduct a quantitative subgroup analysis to determine whether the efficacy of PRP in early stages is superior to that in late stages, as hypothesized.
4. Adverse event reporting: Of the seven included studies, four (57%) failed to provide detailed data on adverse events. This likely reflects incomplete outcome assessment rather than a genuine absence of events. As a result, we are unable to draw definitive conclusions regarding the relative safety profiles of the two therapies.

## 5. Conclusions

Based on the findings of the included studies, PRP demonstrates a statistical advantage in ameliorating HOA symptoms, particularly regarding pain relief at 6–12 months and overall symptom improvement; however, no significant difference was observed in terms of functional recovery. Given the heterogeneity of the current evidence, the risk of bias in certain studies, and the incomplete reporting of safety data, caution is warranted when adopting this modality in clinical practice. Future high‐quality RCTs employing standardized PRP preparation protocols and accounting for pathological staging and individual patient variability are essential to definitively validate the translational clinical value of this therapy.

## Author Contributions

Weisheng Zhuang conceived and designed the study, supervised the project, and critically revised the manuscript for important intellectual content. Di Zhang performed the literature search, study selection, data extraction, statistical analysis, and drafted the manuscript. Shen Hong Ma and He Ling Wang contributed to study screening, data extraction, and prepared Figures 1 and 2. Qiao Hua Han and Kai Shen performed quality assessment of the included studies and prepared Figures 3–8 and Tables 1 and 2.

## Funding

This work was supported by the Science and Technology Research Project of Henan Province (232102310266) and the Medical Science and Technology Research Program of Henan Province (SBGJ202102037).

## Disclosure

All opinions expressed in this article are solely those of the authors and do not necessarily reflect the views of their affiliated organizations, the publisher, editors, or reviewers. The publisher does not guarantee or endorse any product evaluated in this article or any claims made by its manufacturer. All authors have reviewed and approved the final manuscript. All authors agreed to be accountable for all aspects of the work.

## Ethics Statement

The authors have nothing to report.

## Consent

This study is based on publicly available data from published studies, requiring no additional patient consent. No human experiments or private data were involved, and ethical guidelines were strictly followed.

## Conflicts of Interest

The authors declare no conflicts of interest.

## Data Availability

The study’s original contributions are detailed within the article. For additional information, please reach out to the corresponding author.
